# Fabrication of end-coated fibre-optic ultrasound transmitters

**DOI:** 10.1364/BOE.608606

**Published:** 2026-09-08

**Authors:** Semyon Bodian, Shaoyan Zhang, Nyma Nassren, Edward Z. Zhang, Paul C. Beard, Richard J. Colchester, Adrien E. Desjardins, Erwin J. Alles

**Affiliations:** 1 UCL Hawkes Institute, University College London, London, UK; 2 Department of Medical Physics and Biomedical Engineering, University College London, London, UK; 3 Department of Electrical and Computer Engineering, University of British Columbia, Vancouver, British Columbia, Canada

## Abstract

Obliquely emitting fibre-optic optical ultrasound (OpUS) probes show strong potential for guiding minimally invasive procedures, but the imprecise coating methods used to date deposit excess coating along the side of the fibre, which has been suspected to cause imaging artefacts. In this study, we aimed to confirm the source of these artefacts and to introduce an alternative coating method. Optical fibres cleaved at 40° were coated either by conventional dip-coating (DC) or by a new dip-transfer (DT) method, where the curvature of coating droplets allows for accurate alignment of angle-cleaved end-faces. To demonstrate the advantage of DT over DC deposition, fibre-optic ultrasound sources were fabricated using both methods and thoroughly characterised using planar and cylindrical acoustic field scans. Furthermore, a source fabricated with each method was assembled into a fibre-optic OpUS imaging probe and used to image a controlled phantom. DT transmitters generated significantly higher ultrasound pressures than DC transmitters (1.64 ± 0.14 MPa vs 0.53 ± 0.22 MPa, p < 0.05), with comparable mean bandwidths (24.7 ± 1.6 MHz vs 17.3 ± 6.9 MHz) with significantly lower variability. Cylindrical field scans revealed that DC transmitters exhibited strong secondary acoustic lobes (20% of primary beam amplitude), whereas sources fabricated with the DT method did not generate detectable spurious emissions. Imaging trials confirmed that coating of the fibre sides is the primary source of artefacts in obliquely emitting OpUS probes. The DT method effectively eliminates these artefacts while simultaneously enhancing acoustic performance. The higher acoustic performance and the absence of spurious emissions and their associated image artefacts will further advance the quality of OpUS image guidance of minimally invasive interventions. Furthermore, this study reinforces the need for specialised coating methods for obliquely-viewing OpUS probes and highlights the importance the coating coverage in determining image quality.

## 1. Introduction

Ultrasound imaging is a vital tool in guiding minimally invasive surgical (MIS) interventions, which offer shorter and less painful recoveries, lower post-operative infection rates and less scarring compared to conventional open surgery [1–4]. Ultrasound is a desirable modality because it avoids ionising radiation, provides video-rate imaging with high spatial and temporal resolution, and can be implemented using portable equipment [5–10]. Current state-of-the-art miniaturised ultrasound imaging probes for MIS interventions comprise piezoelectric elements [11–14]. However, the small element size ( <0.5 mm) requires delicate dicing and electrical connections, which can be expensive and complicated to achieve [15–17]. In addition, the electrical connections prevent these probes from being used alongside other imaging modalities such as Magnetic Resonance Imaging (MRI) or Computed Tomography (CT), which are commonly employed during MIS interventions, due to their strong interactions with associated electromagnetic fields [18,19].

To overcome these limitations, optical ultrasound (OpUS) generation offers an alternative to piezoelectric ultrasound probes, where pulsed laser light is delivered via miniature (diameter below 0.5 mm) fibre-optics to an optically-absorbing coating to generate ultrasound via the photoacoustic effect [20–22]. Such miniature fibre-optic OpUS imaging has been demonstrated in various MIS configurations, ranging from benchtop and phantom studies to pre-clinical *in vivo* settings [14,23–29].

For efficient and broadband ultrasound generation, OpUS sources are commonly coated with nanocomposites of an elastomeric host doped with an optical absorber. Typically, polydimethylsiloxane (PDMS) is used as elastomeric host due to its high coefficient of thermal expansion (which is directly proportional to the emitted acoustic pressure), optical transparency, and ease of handling [14,24,30–32]. As optical absorbers, carbonaceous materials (e.g. elemental carbon, carbon nanotubes, or candle soot) are commonly used that offer broadband absorption [14,25,30,31,33] and hence allow for a wide range of laser wavelengths.

Various coating deposition techniques have been considered. Dip coating (DC), where the tip of a fibre is manually submerged into a coating solution and withdrawn, is simple and effective but offers little control over the coating thickness [14,23–27]. However, excessive coating thickness introduces significant acoustical attenuation and results in reduced acoustic bandwidth [31]. Candle soot can be directly deposited by placing the fibre end face in a candle flame, and subsequent overcoating with PDMS [25,33]. Electrospinning enables deposition of precisely controlled layer thicknesses, but is only possible for a limited range of materials [34]. Dip coating, direct flame deposition and electrospinning all deposit additional material along the side of the fibre. Micro-inkjet printing has been shown to enable coating deposition on just the fibre end face [23,35], but restricts material choice to low-viscosity formulations, can only be applied on level and straight surfaces, is time-consuming and not readily scalable, and requires specialist equipment.

Previous OpUS imaging probes generated a purely forward (parallel to the fibre long axis) [14,26,29–31] or purely side-emitted (orthogonal to the fibre long axis) acoustic field [36,37]. This directivity was achieved through flat-cleaving optical fibres or deflecting the excitation light sideways using angled reflective surfaces, respectively, and in both cases, excitation light was delivered exclusively to the desired acoustic source area. In contrast, to advance the clinical relevance of fibre-optic OpUS imaging during MIS, recent OpUS sources generate oblique acoustic emissions that offer the partially forward, partially side-viewing functionality required for effective image guidance and navigation [28]. This is achieved by laser cleaving optical fibres at arbitrary angles, which is efficient and scalable and does not require additional structures such as reflectors. However, unlike purely forward or side-viewing alternatives, obliquely emitting OpUS probes require accurate coating of exclusively the angle-cleaved fibre end surface, as excitation light reflecting off the refractive index mismatch between the angled glass-coating interface is delivered to the side of the fibre. If coating material is deposited along the side of such an angle-cleaved fibre, it generates a spurious secondary acoustic emission in an undesired direction that has been observed to generate significant image artefacts [28].

Achieving uniform and spatially confined coatings of carbonaceous-PDMS composite materials on just the fibre end face remains challenging, particularly for the angled and potentially non-planar geometries of laser cleaved, obliquely emitting sources. Here, we propose adopting a dip-transfer (DT) process [38,39] where the fibre is coated using a high-precision manual stage under real-time microscopy image guidance, thus enabling the coating of solely the fibre end-face to avoid spurious additional ultrasound emissions. This accurate deposition approach is expected to result in reproducible coating film thickness and homogeneity. Here, we investigate the DT coating method for reproducibility and uniformity compared with the DC method. The ultrasound generation performances of both methods are assessed, and the directivity of their acoustic emissions mapped. Finally, the imaging performances of OpUS probes comprising a fibre-optic source fabricated using either the DC or DT method are compared in a controlled imaging scenario.

## 2. Methodology

### 2.1. Fibre preparation

Previously presented methods involved lengthy preparation, stripping and cleaving of individual fibres, which is currently employed for the current state-of-the-art deposition methods. Such processing is time-consuming, prone to failure and not readily scalable to become commercially viable. Instead, the proposed method utilises a commercially available laser cutter which enables batches of fibres to be reproducibly cleaved quickly with minimal processing, and can be readily upscaled. The tips of multimode fibres (core diameter: 400 µm, FG400LEA, 0.22 NA, Thorlabs, Germany) were scissor-cut before the fibres were stripped of their buffer coating over a length of 2 cm from the distal fibre tip, exposing the fibre cladding layer, see Fig. 1(a).

**Fig. 1.**
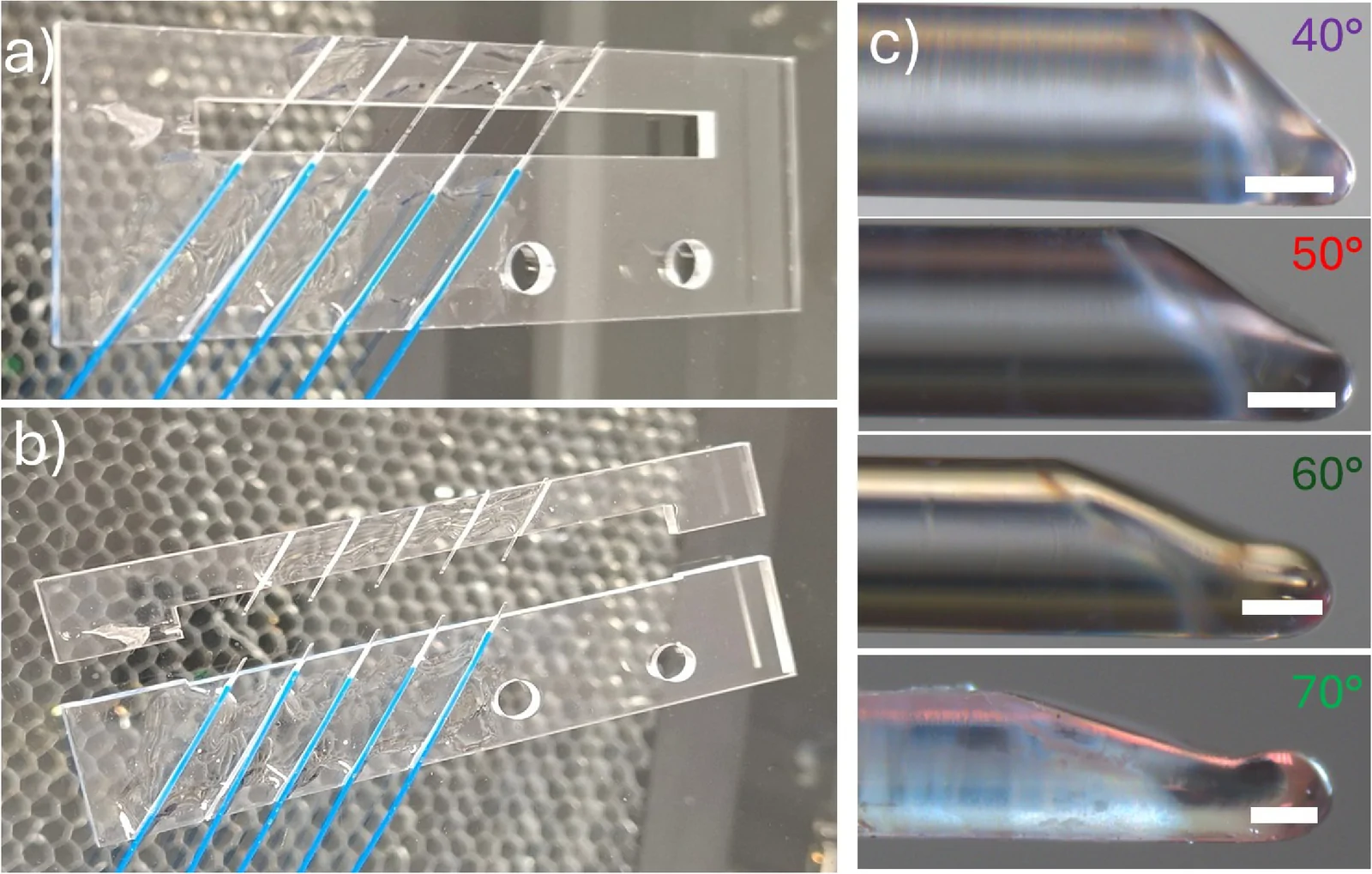
Acrylic mould (left) with fibres glued into angled grooves prior to laser cleaving (a) and following laser cleaving (b). Fibre tips laser-cleaved (c) from top to bottom, at $40^{\circ }$ , $50^{\circ }$ , $60^{\circ }$ , and $70^{\circ }$ . Scale bar: 200 µm.

Batches of five fibres were prepared by placing each fibre in one of the moulds five engraved grooves and secured using a UV-curable epoxy (Norland Optical Adhesive 61, Norland Products, Jamesburg, New Jersey, United States). The mould grooves can be designed and laser-engraved at arbitrary angles, leading to customisable cleave angles; see Fig. 1(c). Each mould contained a slit across which each fibre was suspended when cleaved by the passing laser beam, preventing debris from the mould generated during laser cutting from being deposited onto the cleaved surface. The fibres were cleaved with a laser beam using a commercial laser cutter (Optical power: 60 W, focal spot diameter: 80 µm, power setting: 100%, speed: 2.5%, VLS 4.60, Universal Laser Systems, Scottsdale, Arizona, United States), see Fig. 1(b). Laser cleaving the fibres left each distal end at the same slight incline angle, offset from parallel. This was thought to arise from the nature of the laser beam irradiating through the fibre core. Additionally, the thermal nature of laser cutting and associated melting allowed for the fibre glass to flow, causing deviations from a perfectly planar cleave surface. For large cleave angles, where more heat was deposited to cut along a longer trajectory, more significant surface deformation occurred.

### 2.2. Coating deposition

The coating deposited on the fibre’s distal end enables ultrasound generation via the photoacoustic effect [40,41]. An ideal coating is thin and conformal across the shape of the fibre end-face, providing minimal attenuation to the generated ultrasound which maximises generated pressures and bandwidths [34,42–45]. The coating should also provide full coverage of the fibre end-face and exhibit high optical absorbance at the irradiated laser wavelength to minimise light leakage. Two coating deposition methods were compared. The first is a manual DC approach commonly used in the literature to prepare fibre-optic OpUS transmitters. Here, the fibre tip is submerged within a coating mixture, coating both the fibre tip and sides. The second is a more controlled DT method in which the cleaved end-face of an immobilised fibre is gently brought into contact with a droplet of the coating mixture via a manual stage before being retracted, depositing the coating formulation solely on the fibre-cleaved end-face. While the bulk coating material offers *ca.* 10-15 minutes of meaningful deposition time before noticeably increasing in viscosity, the droplet is replenished after coating every second fibre to minimise changes in the coating solution due to the more rapid solvent evaporation of its small volume. All coated fibres were cured with the fibre long axis upright, in ambient conditions for 24 hours. Five fibres, cleaved at an angle of $40^{\circ }$ , were coated with each of the DT and DC methods.

DT coating was performed under real-time, high-magnification image guidance. A coating station was prepared by affixing the fibre-laden mould to a manual stage with three orthogonal degrees of freedom, and a droplet of coating solution was placed on the top surface of an acrylic platform. The fibre tips were observed under magnification using a CMOS camera (IDS Imaging UI-3240CP-M-GL-TL, Lens system: Navitar Zoom 6000 series, MVL6X12Z, Thorlabs, Germany) and viewed on a computer screen, see Fig. 2, video provided in Visualization 1. Throughout the fibre cleaving, coating and characterisation using field scans, the fibres remained in the moulds to ensure their orientation is fixed.

**Fig. 2.**
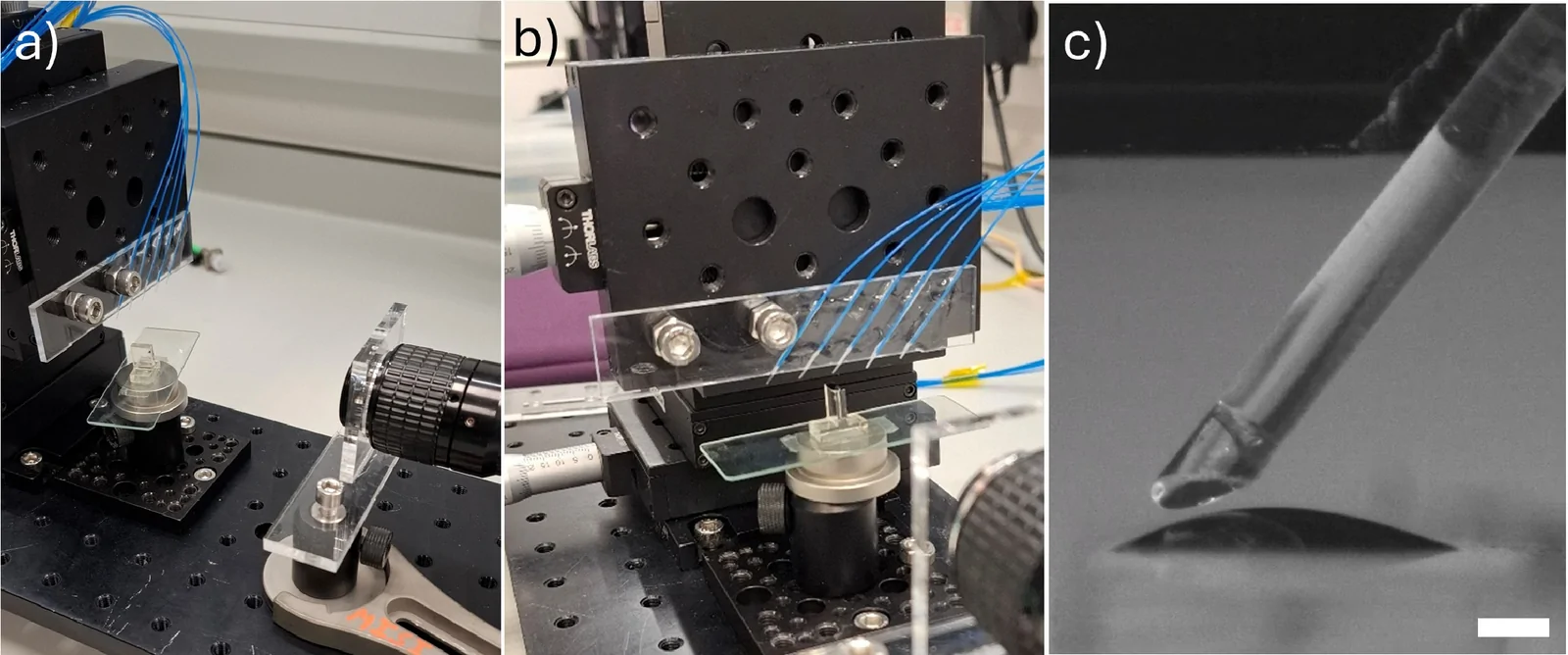
DT coating setup with (a) the CMOS camera aligned with the fibre tips secured into the manual stage via the mould. (b) A side-on view of the fibres positioned above a droplet of carbonaceous coating mixture, (c) with the resulting magnified view provided by the CMOS camera as it appears on the computer screen. White scale bar: 400 µm. A video showing the high-magnification recording of the DT coating process can be viewed in Visualization 1 Visualization 1.

Photoacoustic generating coatings typically comprise a nanocomposite of an optical absorber (to absorb the excitation light) and an elastomeric host (to efficiently convert thermal energy into pressure). Here, PDMS is used as an elastomeric host due to its high coefficient of thermal expansion, and commercially available elemental carbon as a photostable broadband absorber. To prepare the elemental carbon–PDMS composites, a planetary mixer was employed (SpeedMixer DAC 330-100 SE, FlackTek, Landrum, South Carolina, United States). The composites were prepared as follows: 0.20 g of elemental carbon was mixed (750 rpm for 30 seconds, twice, and 2500 rpm for 30 seconds; SKU 699632, Sigma-Aldrich, St. Louis, Missouri, United States) with 4 ml of NeoClear solvent (SKU 1098435000, Sigma-Aldrich, St. Louis, Missouri, United States) [28]. PDMS (Dow-734, Dow Corning, USA) was then combined with the elemental carbon–NeoClear solution in a fixed ratio (0.5 g of PDMS per 1 ml of carbon/NeoClear solution) using the planetary mixer (750 rpm for 30 seconds followed by 2250 rpm for 30 seconds).

### 2.3. Optical absorption

The optical absorption spectra of the coatings were measured over the near-infrared range of 950 to 1200 nm, including the laser wavelength of 1064 nm. Broadband light from a halogen lamp (HL-2000-HP-FHSA, Ocean Optics, USA) was passed through the proximal end of the fibre-optic transmitter and directed towards the coatings at the fibre distal end, which were positioned within an integrating sphere (FOIS-1, Ocean Optics, USA), where the transmitted light was collected. The transmitted light was then recorded by a spectrometer (NIR-Quest512, Ocean Optics, USA, 1000–1800 nm). Reference measurements were obtained by recording the spectra of a bare, uncoated, cleaved optical fibre, and all measurements included corresponding dark measurements to account for background light.

### 2.4. Ultrasound characterisation

The ultrasound generation performance of both types of coatings was assessed using acoustic field scans, from which the generated pressures, bandwidths, beam shapes and divergence were extracted. For both sets of coated fibres, their cleaved end-faces were all offset at the same slight angle to the edge of the mould, which facilitated alignment when coating the end-face, see Fig. 1. Pulsed excitation light (wavelength: 1064 nm, pulse duration: 2 ns, pulse energy: 26.8 µJ, repetition rate: 100 Hz; Q-switched Nd: YAG laser, SPOT-10-500-1064, Elforlight, UK) was delivered through the fibre to the coating deposited at their distal tips, where it was photoacoustically converted into ultrasound.

Planar 2D field scans were performed using a calibrated needle hydrophone (diameter: 200 µm, calibration range: 1 – 30 MHz; NH0200, Precision Acoustics, UK) that was motorized and moved in a grid measuring 3 mm by 3 mm and with a step size of 50 µm (MTS25-Z8, Thorlabs, Dortmund, Germany). The field scan grid was perpendicular to the fibre end-face, with the fibre located at the grid centre, and the hydrophone tip was positioned 1.5 mm from the fibre end-face. Sets of five coated fibres were fabricated using both the DC and DT method, with end-faces cleaved at $40^{\circ }$ , and the field scan recording corresponding to the strongest ultrasound signal recorded for each fibre was extracted to calculate the mean ultrasound pressures and bandwidths (evaluated at −6 dB) for each coating deposition method. To assess whether there were statistically significant differences between these averages, Welch t-tests were performed. F-tests were used to analyse the difference in the variance in the two sets of results, evaluating the reproducibility of each method.

To assess whether additional spurious acoustic emissions are generated, additional 3D cylindrical ultrasound field scans were performed. Here, the fibres were clamped by a pin vice and placed coaxially within a rotational stage, orthogonal to the needle hydrophone. The hydrophone was translated along an 8 mm long aperture parallel to the optical fibre in steps of 50 µm, and the fibre was rotated across a full $360^{\circ }$ revolution in increments of $1^{\circ }$ , see Fig. 3.

**Fig. 3.**
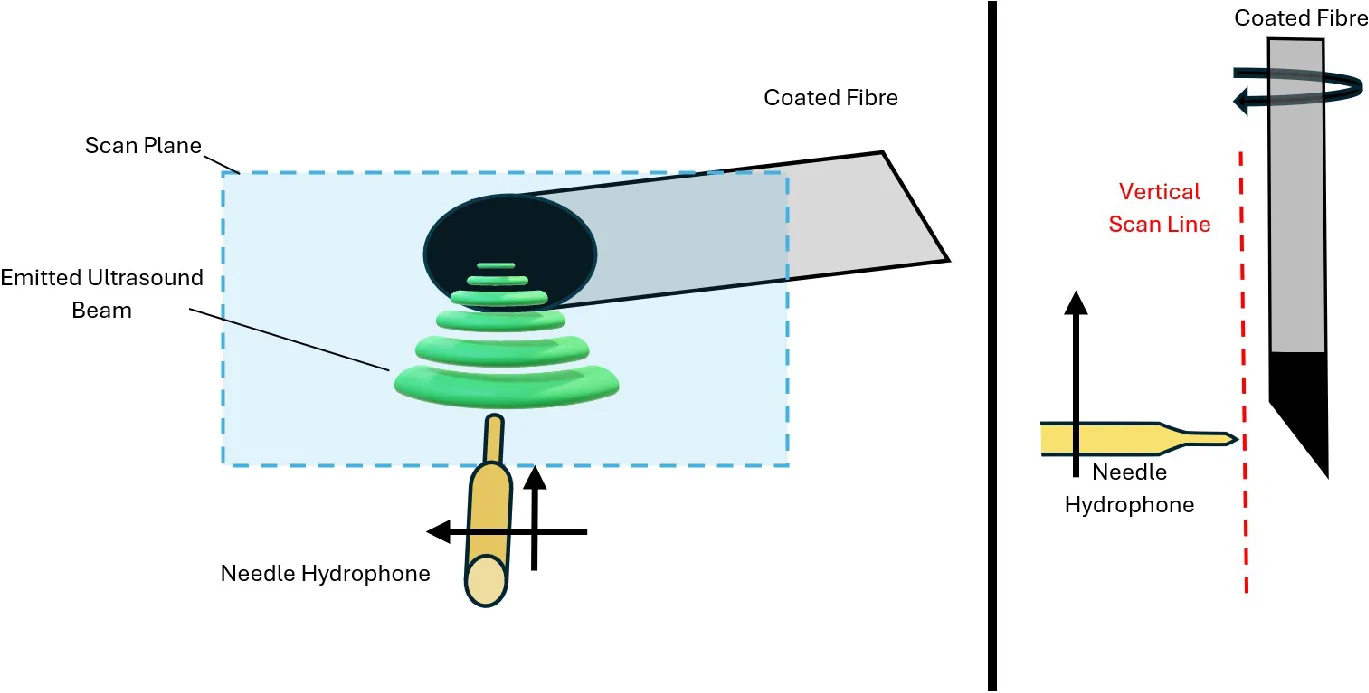
The acoustic performance of both coated fibres was assessed across both a planar, rectangular field scan oriented parallel to the angle-cleaved fibre end-face (left) and a cylindrical surface around the fibre long axis (right). Arrows on both the needle hydrophone (left and right) and cleaved fibre (right) indicate their motion during the respective field scans.

### 2.5. Pulse echo ultrasound imaging

One fibre-optic ultrasound source of each DC and DT design was paired with a fibre-optic ultrasound detector comprising a Fabry-Pérot cavity [46]. The optically resonant Fabry-Pérot sensor was interrogated by continuously monitoring its reflectivity, which is modulated by impinging pressure waves, using a wavelength-tunable laser (wavelength: 1500–1600 nm, power: 5 mW, Tunics T100S CL, Yenista Optics, France) tuned to the sensor bias point and a custom photodiode [28]. The detector was carefully aligned to position the sensitive element at the apex of the angle-cleaved surface of the source, see Fig. 4, and fixed in place using a cyanoacrylate superglue adhesive (SuperGlue 3G, Loctite, Henkel, Germany).

**Fig. 4.**
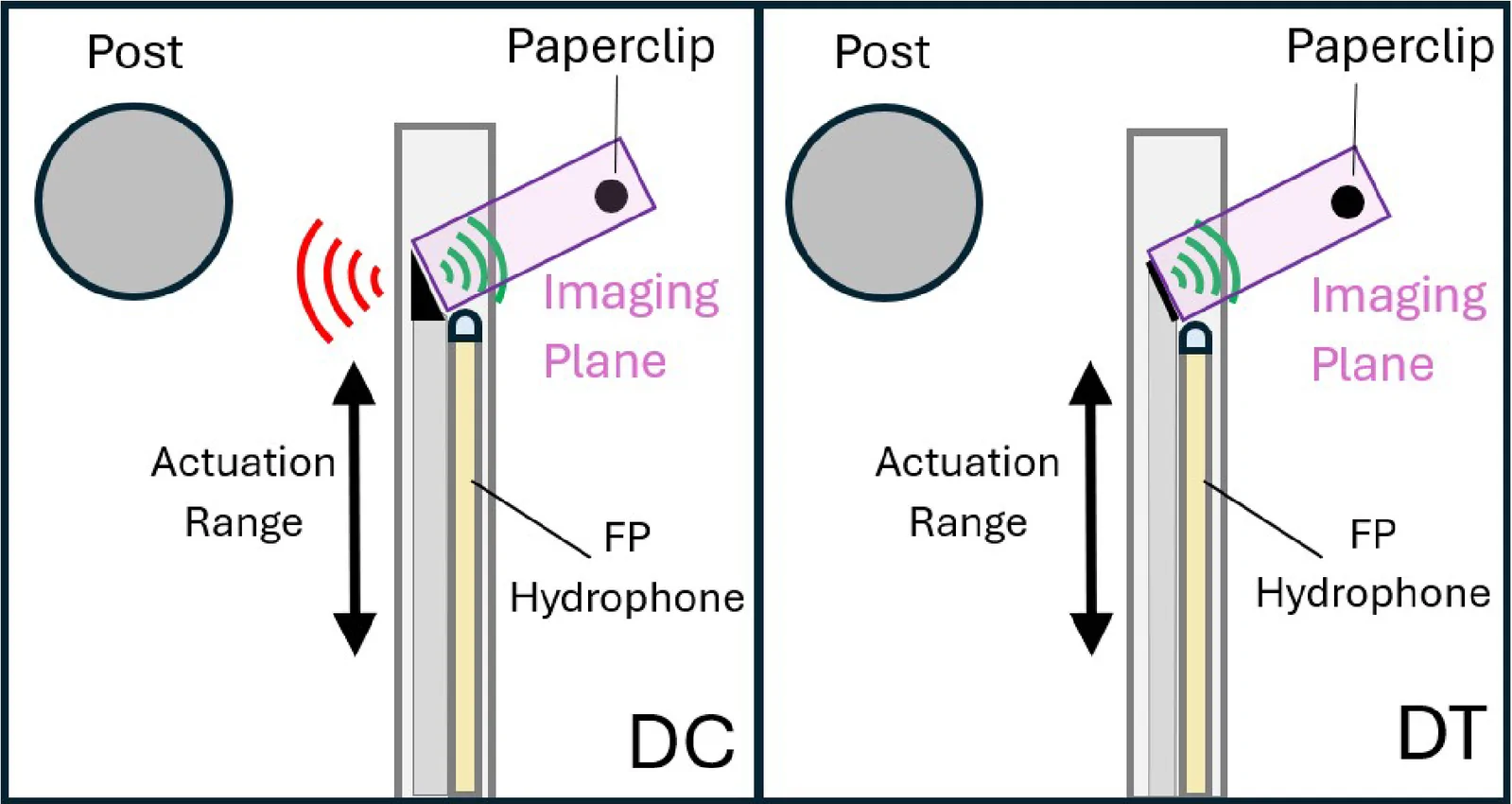
Imaging set-up for the pulse-echo ultrasound imaging using transmitters fabricated using the DC (left) and DT (right) methods. Transmitters are paired with Fabry-Pérot fibre-optic sensors for detection of pulse-echo signals. The imaging scenario comprised a wire target (black dot) positioned on the right (representing the desired imaging target) and a metal post positioned on the left (placed opposite to the desired imaging direction). The actuated motion of the fibre is highlighted in black and the resulting imaging plane is highlighted by the purple area (not to scale). The desired primary ultrasound signal is shown in green with the spurious emission is indicated in red.

To verify the artefact reduction achieved through the DT transmitter approach, comparative imaging experiments were performed on an identical target using both a DC and a DT OpUS transmitter. For the experiments, a probe incorporating a transmitter was enclosed within an immobilised ultrasonically transparent EBUS sheath. A steel wire (diameter: 0.78 mm, placed orthogonal to the image plane, 7 mm from the probe) acted as an imaging point target, representing the desired signal. A metal post (diameter: 25 mm) was placed on the opposite side at a distance of 13 mm from the imaging probe. In an ideal case where the imaging probe only emits ultrasound in the oblique direction, this metal post should not generate pulse-echo signals, see Fig. 4. Image acquisition was performed by continuously actuating the OpUS probe linearly over a 30 mm-wide aperture (from 0 to 30 mm) in a back-and-forth motion, resulting in a frame rate of 4 Hz. During actuation, pulse-echo signals were continuously acquired, collated, and reconstructed into images.

The received pulse-echo signals underwent amplification by 30 dB before being processed and band-pass filtered between 1.5 and 12 MHz. This results in improved spatial resolution following delay-and-sum (DnS) reconstruction [47]. The lower frequencies ( 0−1.5 MHz) were omitted as neither DC nor DT sources generated meaningful acoustic power across this bandwidth. The higher frequencies ( >12 MHz) were suppressed as at these frequencies the OpUS source geometry was substantially larger than the acoustic wavelength, and the corresponding source directivity rendered the DnS algorithm – which assumes omnidirectional sensitivity – ineffective across these frequencies. Artefacts arising from direct acoustic cross-talk between the transmitter and receiver and from reflections within the weakly reflective protective sheath were suppressed by singular value decomposition filtering [48]. Specifically, the filtering removes components associated with large singular values, which correspond to signals that are highly correlated in both space and time. Time-gain compensation was performed, followed by Hilbert transform-based envelope detection. Afterwards, a logarithmic compression was applied. The same imaging process was subsequently repeated by withdrawing the DC probe from the immobilised sheath and inserting a DT probe, ensuring that the target location and orientation remained identical.

## 3. Results

### 3.1. Source fabrication

The end-on microscope images of the DC coatings show the coating covering the entire fibre end-face and sides. The corresponding end-on microscope images of the DT coatings show a homogeneous black coating with a grainy appearance that becomes slightly sparser at the edges of the fibre, see Fig. 5. Overall, the fibre sides remain completely uncoated with the coating covering the majority of the fibre end-face. The microscope images of the coatings from a side-on view reveal a thin coating which conforms very closely to the cleaved face. Measuring a coating’s thickness is very challenging since the coating is highly optically absorbing and the fibre is optically transparent. Thus, we cannot measure the coating thickness using microscope images of the fibre prior and post-coating. Both DC and DT coatings exhibit a shiny exterior and a textured surface.

**Fig. 5.**
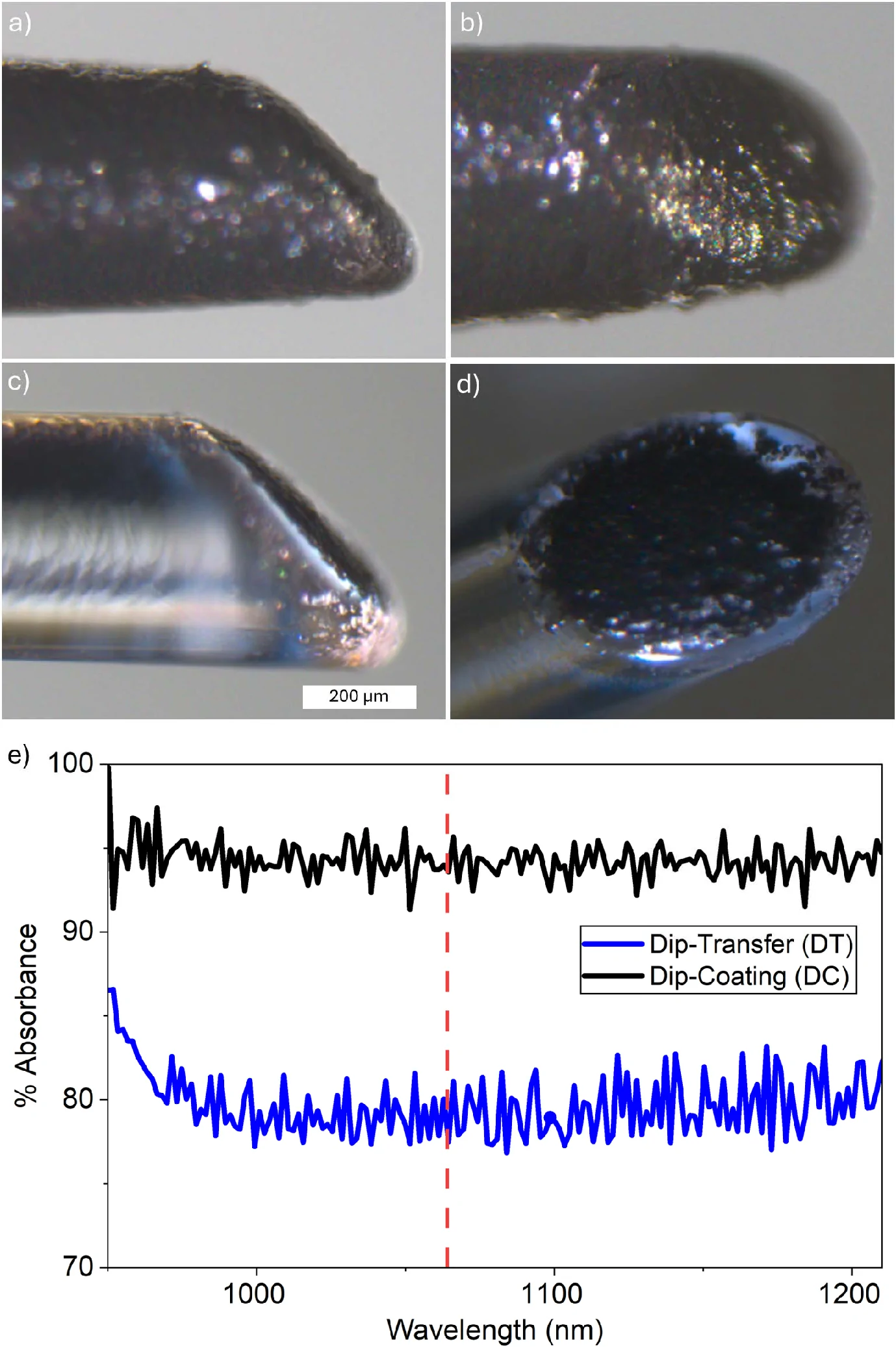
Microscope images of optical fibre tips coated using the DC (a, b) and DT (c, d) methods with side (a, c) and end-on views (b, d). Absorption spectra (e) of DC (black line) and DT (blue line) with the percentage absorbance graphed against wavelength. The 1064 nm wavelength, used for photoacoustic ultrasound generation, is signified by the red dashed line.

### 3.2. Optical absorption

The absorption spectra of the DC and DT fibres are shown in Fig. 5(e). At the 1064 nm wavelength generated by the pulsed laser used for ultrasound generation, the DT fibres demonstrated an optical absorbance of 79% whilst the DC fibres had a higher optical absorbance of 94%. The lower optical absorbance of the DT fibres compared to the DC fibres arises from light being internally reflected within the DT fibres rather than being absorbed by the side coating, as in the case of the DC fibres. Thus, less light reaches the coated fibre-end face of DC fibres than that of DT fibres and thus the former records a higher optical absorption. In comparison, AiO-coated CSNP-PDMS fibres demonstrated optical absorbances of above 99% [25]. Despite a small spike in absorption at 950 nm, the DC fibre absorption spectrum is relatively constant over the observed range of 950 nm to 1200 nm, averaging around 95% absorption. The corresponding DT fibre absorption spectrum shows a more pronounced dip from 950 nm to 975 nm, decreasing from around 87% to 80%. The spectrum slightly increases with wavelength from 975 nm to 1200 nm.

### 3.3. Acoustic characterisation

The average ultrasound pressure measured for fibre-optic ultrasound sources comprising DT coatings was 1.64 ± 0.14 MPa (mean ± standard deviation), while DC coatings exhibited a lower average of 0.53 ± 0.22 MPa. A Welch’s t-test revealed a statistically significant difference between the two groups (t = 9.55, p < 0.0001). To determine whether the choice of coating strategy affected the variability of acoustic performance across the repeats, an F-test was used to analyse the variance of each ultrasound measurement. In particular, the variance of the measurements indicates the reproducibility of each method. The F-test for variance revealed an F-value of 2.54 (p > 0.05), indicating that the difference in variability between DT and DC coatings for ultrasound pressure was not statistically significant. Given how both DC and DT sources used identical fibre type, cleave angle, coating material recipe, and optical excitation parameters, any differences in acoustical performance are likely due to the coating morphology.

DT coatings demonstrated a more consistent bandwidth with an average of 24.7 ± 1.6 MHz compared to the more variable bandwidth of DC coatings (17.3 ± 6.9 MHz). Although the mean bandwidth recorded by the DT coatings was wider than that measured from the DC coatings, the difference was not statistically significant. This is shown by the Welch’s t-test for mean difference which yielded a p-value of approximately 0.072. To evaluate reproducibility, an F-test for variance comparison gave an F-value of 18.31 (p = 0.0155), indicating a statistically significant difference. This result confirms that DT coatings are statistically significantly more consistent in their emitted acoustic bandwidth than DC coatings. This finding demonstrates the greater control the DT process provides when applying the coating to the cleaved fibre end-face, resulting in more consistent, thinner coatings that lead to higher pressures and comparable bandwidths (Table 1).

**Table 1.** Table displaying ultrasound pressures (MPa) and bandwidths (MHz) recorded from five replicates each of the DC and DT transmitters along with their respective means. Alongside each mean is its standard deviation (S.D.), as well as p -values indicating the degree of statistical significance between each of the two averages for each measurement.

	DC							DT							
	
	1	2	3	4	5	Avg.	S.D.	1	2	3	4	5	Avg.	S.D.	p
Pressures (MPa)	0.41	0.26	0.65	0.48	0.83	0.53	0.22	1.51	1.51	1.63	1.84	1.69	1.64	0.14	0.00004
Bandwidths (MHz)	15.8	10.6	12.9	19.0	28.2	17.3	6.9	22.6	25.9	25.4	23.4	26.2	24.7	1.6	0.072

### 3.4. Directional acoustic emissions

The cylindrical field scan of the DC fibre reveals an ultrasound signal generated in two opposite directions from the fibre. The spurious emission magnitude measured around 20% of that of the main beam. The DT fibre’s equivalent scan only revealed that of the desired oblique ultrasound emission towards a single direction, and no spurious emission was observed, see Fig. 6. Due to difficulties in aligning the hydrophone relative to the rotational mount, the cylindrical field scans were acquired at different diameters (6 mm vs. 4 mm for DT and DC, respectively). Numerically correcting for the accompanying geometrical attenuation was not possible due to the broad bandwidth and frequency-dependent directivity resulting from an extended and slightly non-planar source surface. Consequently, quantitative comparison of the pressures and bandwidth was only possible for the planar field scans.

**Fig. 6.**
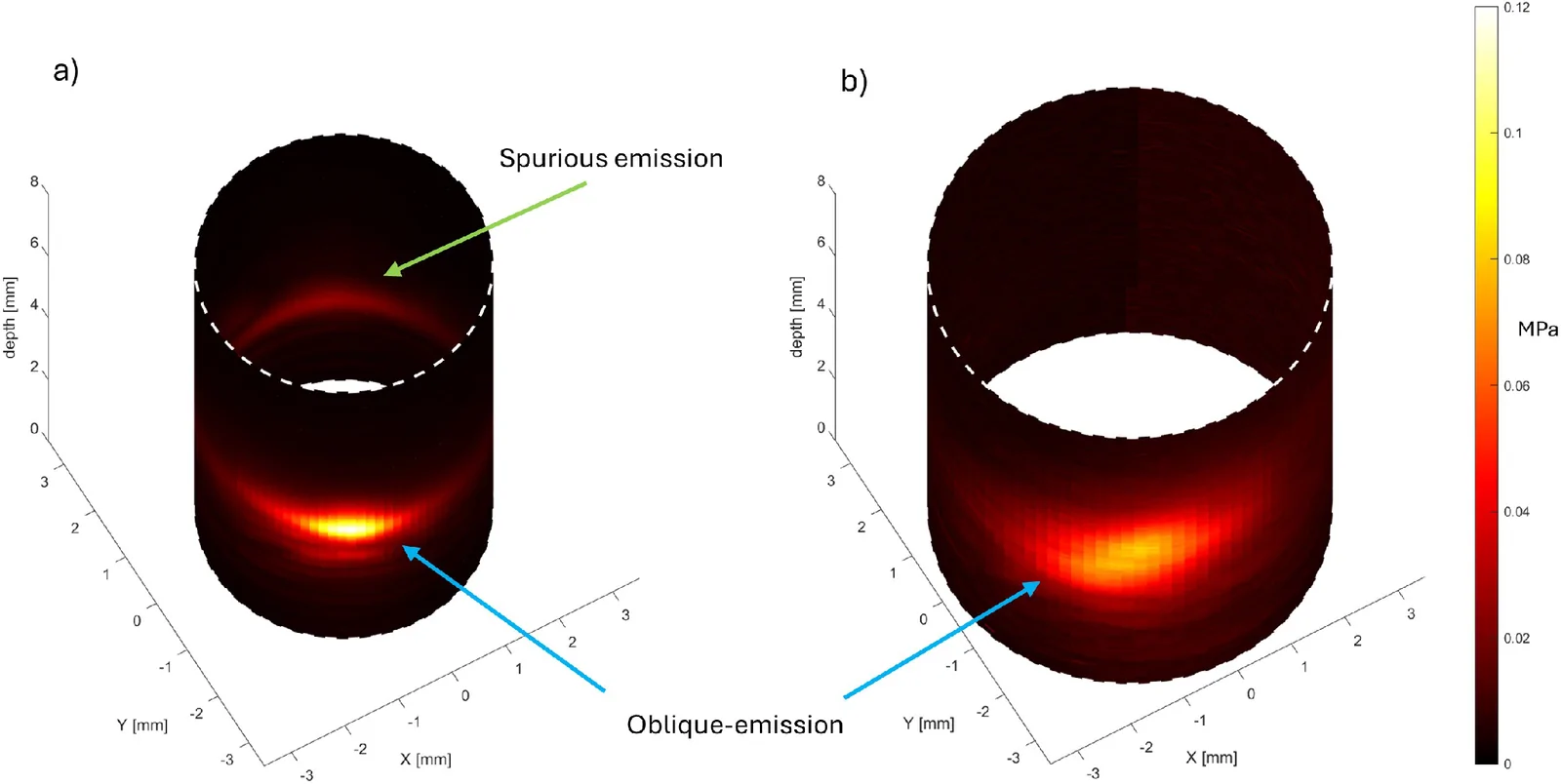
Cylindrical field scans of the DC (a) and DT (b) fibre-optic transmitter with the oblique and spurious ultrasound signals indicated by the blue and green arrows, respectively. A colour bar on the right indicates the ultrasound pressures (MPa) generated by each signal.

### 3.5. Imaging performance

Pulse-echo ultrasound B-mode images were acquired using two distinct fibre-optic OpUS imaging probes, featuring either the DT or the DC transmitter. Both recorded images exhibit a high-amplitude point-like target at an axial depth of 7 mm, with a crescent-shaped artefact due to spatial subsampling and acoustic ringing within the highly echogenic metal wire. The lateral position of this feature shifted from 15–20 mm in the DC-transmitter image to 20–25 mm in the DT-transmitter image.

The DC-transmitter image revealed a secondary discrete feature (highlighted by the green square) at an axial depth of 20–25 mm. Despite a lower pressure emitted in this spurious direction and a larger distance to the object, the image of the metal post exhibits an amplitude similar to that of the actual imaging target. This is attributed to the specular nature of the pulse-echo reflection off the highly echogenic post combined with the normal incidence of the spurious emission, and represents a ‘worst-case’ scenario. Nevertheless, artefacts of significant amplitude have previously been observed even when imaging tissue-mimicking material using a DC-style probe [28]. Notably, this feature was absent in the DT-transmitter image. Both images are characterised by a high contrast-to-noise ratio, with negligible background speckle or electronic noise artefacts.

A small signal is observed at an axial depth of 13 mm in the DC-transmitter image which corresponds to the air-water surface of the water bath given that it matches the probe’s depth in the water bath. This feature is substantially weaker for the probe comprising a DT source, further confirming how accurate coating of just the angled source surface reduces image artefacts.

## 4. Discussion

This study compared the DC and DT methods and assessed whether the former could selectively coat the fibre-cleaved end-face, aiming to avoid image artefacts observed in previous imaging studies of obliquely emitting fibre-optic OpUS probes [28]. Upon confirmation of end-face-coated transmitters, it was evaluated whether the DT coating strategy eliminated the imaging artefacts observed in previous obliquely emitting fibre-optic OpUS probes prepared using the DC method.

The DT method selectively coated only the fibre-cleaved end-face by controlling fibre orientation, descent, and removal during contact with the coating solution, whereas the DC method coated both the end-face and the fibre sides. Similar coating homogeneity in both cases suggests that local coating uniformity was mainly governed by coating preparation rather than the deposition method. Cylindrical field scans showed that coating of the fibre sides in DC transmitters produced spurious ultrasound emissions, while DT transmitters generated a single beam. This was confirmed by B-mode imaging, where DT transmitters avoided the characteristic artefacts observed with DC transmitters, improving image fidelity.

Light leakage from the uncoated fibre sides was most evident in the DT fibre, which had lower optical absorption (79%) than the DC and AiO CSNP-PDMS coatings (94% and 99%, respectively). A reflective material, such as chrome paint, could be applied to the fibre sides to avoid light leakage and avoid intense illumination of the fibre surroundings, or even reflect this light back to the coating to further enhance ultrasound generation. Such a reflective coating could be deposited prior to laser cleaving to ensure coverage of just the fibre sides, or deposited after DT coating of the OpUS generating material by either “painting” the fibre sides or over-coating the DT-coated fibre with reflective material, provided the reflective layer is sufficiently thin to be acoustically invisible. Both DT and DC coatings also generated lower ultrasound pressures and narrower bandwidths than the AiO CSNP-PDMS coatings. Light leakage is an issue because, firstly, it leads to sub-optimal coupling of laser pulse energy into the end-face coating, impairing the generated ultrasound pressures and bandwidths. Secondly, for the proposed application of guiding MIS interventions, any leaked light could affect the surrounding tissue during *in vivo* use, raising safety concerns. Finally, leaked light could potentially generate photoacoustic signals within the tissue, creating image artefacts.

PDMS has been reported to exhibit weak methyl (C-H) vibrational overtone absorption near 910 nm, with the third overtone methyl (C–H) stretching band experimentally observed at approximately 908 nm [49]. It is therefore possible that the tail of this peak accounts for the higher absorption at 950 nm than at 975 nm, resulting in the dip observed in the DT fibre spectrum. The DC fibre also shows a higher absorbance at 950 nm, but the decrease at larger wavelengths is less pronounced compared to that seen in the spectrum of the DT fibre. This could potentially be because less light reaches the DC coating.

The DT method has demonstrated that it can produce pressures up to three times those of the DC method. There was no statistically significant difference in the variability of the pressures generated between the two sets of fibre-optic transmitters. The statistically significantly higher ultrasound pressures generated by the DT fibres than the DC equivalents may be attributed to a lower coating thickness, enabled by greater control over coating the fibre end-face. Specifically, less coating solution is exposed to the fibre end-face than in the DC method, where the fibre end-face is completely submerged, resulting in a larger initial coating deposit. With thicker coatings, one expects greater acoustic attenuation, reducing the magnitude of the generated ultrasound pressure [23,31].

Note that in this study the coating thickness could not directly be measured, and hence the reported lower DT coating thickness relative to that achieved via DC was merely inferred from visual observation (*cf*. Fig. 5) and acoustical performance. Measuring the coating thickness directly is difficult because the coating is optically absorbent across a broad optical bandwidth. Optical microscopy is insufficient due to a lack of visual contrast or visibility, particularly when both the end and sides of the fibre are coated as in the case for DC coatings. Other optical methods such as optical coherence tomography or white light profilometry are unsuitable because of the high optical absorbance of the coating and the corresponding limited penetration. Scanning electron or atomic force microscopy could reveal the coating surface morphology, but are not capable of also visualising the subsurface fibre end face. This fibre end face is known to be slightly non-planar (*cf*. Figure 1) and hence would require detailed mapping for accurate thickness measurements – likely requiring surface profiling both before and after coating deposition.

The bandwidths generated by the DT method were wider than those produced by the DC method, but the difference was not statistically significant. This aligns with the assumption that the DT coatings were thinner than the DC coatings. This would mean that higher ultrasound frequencies generated by the DC coatings experience greater acoustic attenuation than those produced by the DT coatings. Furthermore, the DT method produced coatings with greater reproducibility, reinforcing the claim that it produces more consistent coatings than the manual DC method. However, in this study just five sources were fabricated and characterised using both the DC and DT approaches, and using larger groups would be required to establish batch-to-batch reproducibility and generalisability. Overall, using a laser cutter to cleave the fibres offers a reproducible method for achieving clean, consistent end-faces on multiple fibres in a single fabrication step. Arbitrary cleave angles can be achieved by designing moulds with grooves whose angles match those of the desired cleaves and thus, this method can be used to design fibre-optic transmitters custom-made to guide specific clinical interventions. The method employs a commercially available laser cutter and requires no fibre preparation beyond stripping the fibres’ distal ends and immobilising them within the moulds with a UV-curable adhesive. The size of this setup could easily be increased to cleave more fibres per batch. It is worth noting that, despite the use of an accurate micropositioner, the DT method still involves a large number of variables to control. For instance, factors such as the rate of fibre retrieval from the coating droplet are difficult to standardise using manual control. In addition, when coating a fibre, the location where the fibre contacts the coating droplet determines which part of the fibre end-face is coated. Positioning the angle-cleaved fibre end-face parallel to the local droplet curvature may minimise any coating of the fibre sides. In future experiments, a motorised withdrawal stage, combined with a closed-loop feedback approach based on computer vision, could be used to fully automate the DT process and further improve reproducibility.

Furthermore, other factors affecting the droplet can influence the resulting coating. For instance, it could be assumed that the coating droplet’s viscosity is related to its size and thus perhaps using a larger droplet would lead to a larger degree of inflection when the fibre is brought into contact with it. This could then lead to more coating on the fibre sides. In addition, larger droplets contain more solvent, resulting in a smaller relative solvent loss and therefore a more constant viscosity over time. If the coating solution is not fully homogeneous, the origin of the droplet within the solution can also influence the morphology and coverage of the fibre coating. For example, a droplet drawn from the bottom of the solution may contain a higher concentration of elemental carbon nanoparticles than one taken from the surface. Going forward, these variables will need to be investigated to further optimise the DT coating methodology. This could also include the use of an automated stage, whose approach to and retraction from the coating droplet could be even better controlled, possibly resulting in more consistent coatings.

## 5. Conclusion

This study confirmed that coating of the fibre sides during DC is the source of the spurious ultrasound emissions responsible for the image artefacts previously observed in obliquely-emitting fibre-optic OpUS probes. The newly developed DT method is a three-step process where multiple OpUS transmitters are fabricated simultaneously, offering both an ease of fabrication and the potential for the process to be scaled up to coat more fibres in one batch (Fig. 7). In addition, immobilising the fibres in moulds before cleaving and coating increases method consistency across the batch. Moreover, using precise, controllable stages under magnified observation further enhances the method’s reliability. The DT fibres generated higher, statistically significant ultrasound pressures than DC fibres, along with wider ultrasound bandwidths and a statistically significantly lower variance. These findings show great promise for future clinical and commercial translation of these optimised OpUS transmitters. The novel coating deposition strategy for fibre-optic OpUS sources presented here will further advance the quality of OpUS image guidance of minimally invasive interventions.

**Fig. 7.**
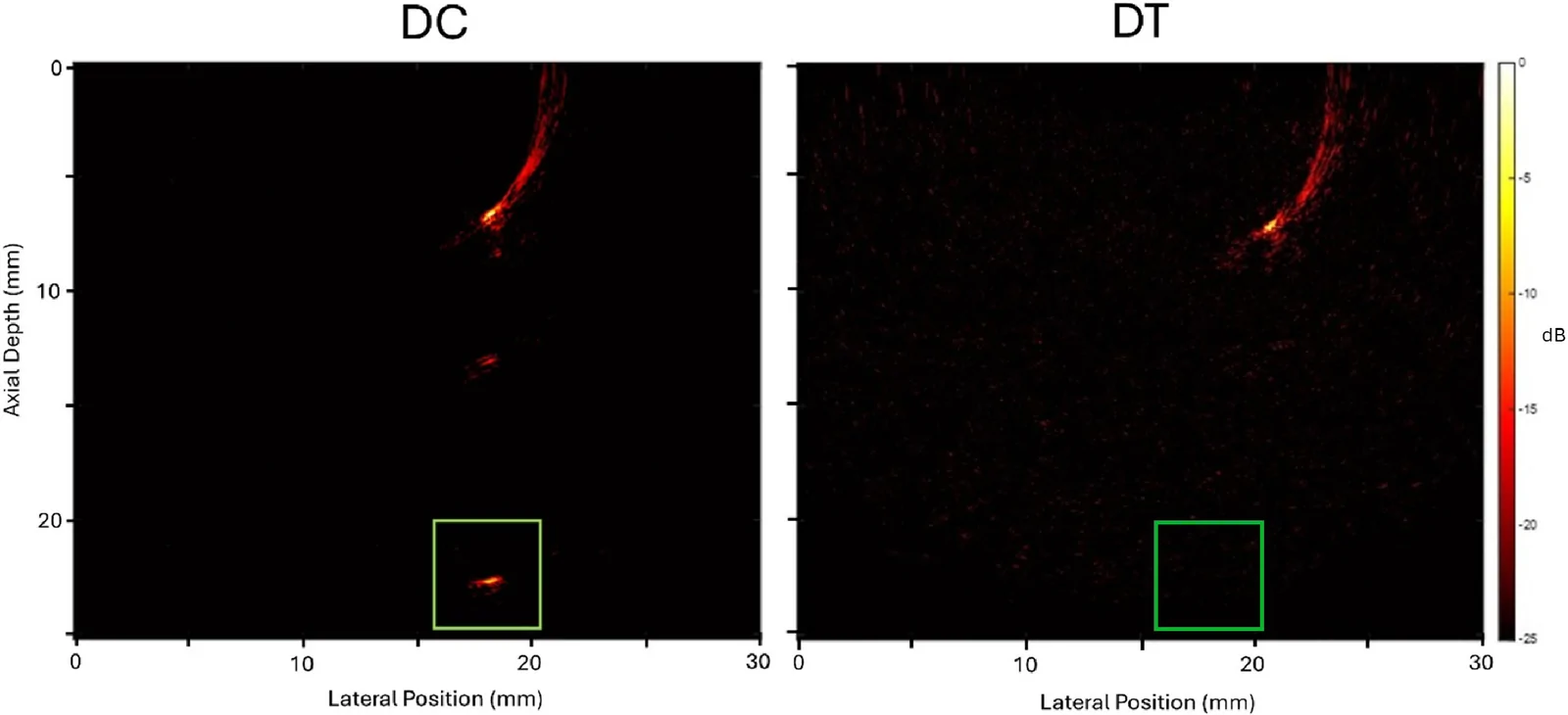
Pulse-echo B-mode images of fibre-optic OpUS imaging probes featuring DC-transmitter (a) and DT transmitter (b) positioned between an optical post and a paperclip. The green box highlights the signal produced by the spurious emission’s reflection off the metal post in the image produced with the DC-transmitter and its subsequent absence in the image produced using the DT-transmitter.

## Supplemental information

Visualization 1 A video showing magnified view of Dip Transfer fibre coating method. https://doi.org/10.6084/m9.figshare.32744511

## Data Availability

The data that support the findings of this study are available from the corresponding author upon reasonable request
